# Approaches, achievements, challenges, and lessons learned in setting up an urban-based Health and Demographic Surveillance System in South Africa

**DOI:** 10.1080/16549716.2021.1874138

**Published:** 2021-02-03

**Authors:** Sunday A. Adedini, Dineo Thaele, Matshidiso Sello, Portia Mutevedzi, Cleopas Hywinya, Nonhlanhla Ngwenya, Nellie Myburgh, Shabir A. Madhi

**Affiliations:** a Medical Research Council: Vaccine and Infectious Disease Analytics Research Unit, Faculty of Health Sciences, University of the Witwatersrand, Johannesburg, South Africa; b Department of Science and Technology/National Research Foundation: Vaccine Preventable Diseases Unit, Faculty of Health Sciences, University of the Witwatersrand, Johannesburg, South Africa; c Programme in Demography and Population Studies, University of the Witwatersrand, Schools of Public Health and Social Sciences, South Africa

**Keywords:** Stig Wall, Umeå University, Sweden, Public health, demographic surveillance, child health, urban, south Africa

## Abstract

Reliable civil registration and vital statistics (CRVSs) are essential for estimating mortality rates and population changes, and are critical for public health and socio-economic planning. CRVSs are largely incomplete in Africa, thus Health and Demographic Surveillance Systems (HDSSs) fill gaps in CRVSs, albeit existing HDSSs in South Africa are in rural areas. This limits the generalisability of such data in a country such as South Africa where over 60% live in urban areas, and where there are limitations to access health and social services. We describe the approaches, achievements, challenges and lessons learned in setting up a HDSS site in Soweto and Thembelihle (SaT-HDSS), Johannesburg; which is the first urban-based HDSS in Southern Africa. We also highlight a number of studies being implemented in the HDSS. In 2017–2020, the HDSS has enrolled 124,169 individuals and followed up 95% of this population through 3 rounds of data collection. Several challenges were encountered during the initiation of the HDSS, including difficulties in community mobilisation and entry, stakeholders’ engagement and participation, inaccessibility problems and concerns about safety of fieldworkers, and difficulty in getting/recruiting technical staff with requisite experience. Nevertheless, the SaT-HDSS was successfully established through application of several strategies, including continuous community engagement and stakeholders’ mobilisation; in-depth training and retraining of all study staff; technical support from well-established HDSS sites across Africa, and international academic collaborations. Despite the challenges of undertaking routine surveillance of a hard-to-reach and highly mobile population, the SaT-HDSS was successfully established with a high-retention rate. The HDSS offers an important lens on morbidity and mortality and serves as a platform for pilots of interventions and programmes aimed at improving health and well-being of an urban population.

## Background

Timely and high-quality data are critical for monitoring a country’s performance towards achieving the Sustainable Development Goals (SDGs). Although socio-economic and public health policies and programmes are often designed with a set of indicators to monitor progress, relevant data for measuring performance are not always available in many countries. Policymakers and programme implementers in many low- and middle-income countries (LMIC) often do not have access to essential data for evidence-based socio-economic and public health planning. In particular, while complete and accurate records of civil registration and vital statistics (CRVSs) are useful for mortality estimations [1–3], many countries in sub-Saharan Africa (SSA) and South Asia have inadequate vital registration systems [4]. To bridge this gap, more than 45 health and demographic surveillance system (HDSS) sites that collect routine data on demographic and health issues have been established across SSA and South Asia, with over 3 million people under surveillance [5]. The majority of these sites are, however, rural-based with only around 10 being in urban settings. In South Africa, where over 60% of the population live in urban areas, all three HDSS sites established between 1992 and 2000 are in rural settings, despite the increasing rate of urbanisation.

With two in every three South Africans residing in urban areas [6], the high rate of urbanisation has continued to strain the capacity of city authorities to provide essential basic services such as housing, potable water, healthcare, education, security and sanitation. Consequently, a high proportion of urban residents live in slum or slum-like areas [7–9], leading to urban poverty or deprivation. This contributes to high rates of crime, unemployment, malnutrition, poor health outcomes, and high risks of morbidity and mortality among children and adult population. Addressing this complex set of problems requires timely and high quality empirical population level data.

While the urban population may have greater access to basic amenities and vital registries (for registration of births and deaths) than the rural residents, increasing number of urban residents are facing significant limitations to access health and social services. Thus, HDSS data can provide important evidence-based insights into the health and socio-economic challenges facing the increasing number of urban population. Through routine data collection, HDSS can provide details on the constantly changing pattern of socio-economic and health situations in urban areas which may be difficult to obtain through censuses and periodic surveys. Previous experience has demonstrated that population health perspectives through HDSS platforms provide timely information that is required for evaluation of interventions on health behaviours and outcomes [10]. Hence, an urban-based HDSS will serve as important research platform for impact evaluation and intervention studies among the urban population.

We established an urban-based HDSS in Soweto and Thembelihle as a component of an international programme on child health entitled – Child Health and Mortality Prevention Surveillance (CHAMPS). Detailed description about CHAMPS has been reported elsewhere [11,12]. The HDSS lends itself to providing evidence on broader population and public health issues. This paper describes the approaches, achievements, challenges and lessons learned in setting up the urban HDSS site in a low-middle-income setting in Soweto and Thembelihle and its surrounding informal settlements in Johannesburg South, Gauteng Province, South Africa. The remainder of this paper is structured as follows: The method section presents the aim of SaT-HDSS, selection of study area, data elements, data collection approaches and data quality. The result section presents some key demographic indicators collected in the HDSS. The discussion highlights the major challenges encountered while implementing the HDSS. The discussion section also presents the strategies employed in addressing the challenges as well as lessons learned in setting up the HDSS.

## Methods

### Aim of SaT-HDSS

The SaT-HDSS was established in late 2017 as part of the CHAMPS programme. CHAMPS is a multi-country programme that seeks to track the definitive causes of child death in sub-Saharan African and South Asia. The HDSS was set up as one of the three components of CHAMPS programme. The other two components of CHAMPS are the Social Behavioural Sciences (SBS) and the Minimally Invasive Tissue Sampling (MITS) projects. All the three components were established to contribute to the achievement of the overarching goal of the CHAMPS programme – which is to prevent childhood deaths and illnesses through the provision of timely data for a better understanding of the causes of child death in SSA and South Asia.

The HDSS, as a component of the CHAMPS programme, was set up to provide population level data for estimating rates of adverse pregnancy and child health outcomes. The initial focus was on stillbirths and under-5 mortality coupled with biological investigation of the deaths using the MITS procedure to provide details on the causes of these events [11]. Data on individual, household, and community factors associated with mortality are collected at SaT- HDSS as part of the CHAMPS programme.

Using longitudinal follow-up of the households and members, the SaT-HDSS collects information for assessing behavioural, health systems, environmental, and geographic contributors to adverse pregnancy outcomes and child mortality. As shown in Table 1, data are collected in the HDSS (as part of the CHAMPS programme) using three levels. Furthermore, the HDSS is used for nested studies and intervention programmes aimed at improving health and well-being of the population under surveillance

**Table 1.** Data elements in Soweto and Thembelihle Health and Demographic Surveillance System

Level of data elements		Description
**Level 1**		The core indicators which are essential towards meeting the CHAMPS objectives. They include age- and sex-specific population size, numbers of births and deaths by age and sex, in-migration and out-migration.
**Level 2**		Covers the household and individual characteristics (which are important for contextualising CHAMPS results) including: Household socioeconomic status; access to water, electricity and sanitation; cooking fuel; access to healthcare; and living arrangement. Mother characteristics – age, education, marital status, religion, and reproductive history. Children characteristics: antenatal care, delivery, postnatal care, immunization uptake and breastfeeding.
**Level 3**		Involves enhanced data collection modules that focus on health topics, including biomarkers such as anthropometric and haemoglobin measurements, nutrition assessment, HIV screening and testing, COVID-19 screening, testing and research; as well as food frequency questionnaires or 24-hour dietary recall.

Abbreviation: CHAMPS – Child Health and Mortality Prevention Surveillance COVID-19 – coronavirus disease 2019

### The study area

Establishment of the SaT-HDSS was initiated in November 2017 in Soweto, a low-middle income township spanning an area of 200 km^2^, approximately 20 km south-west of Johannesburg. Soweto is inhabited mainly by black-Africans, with an estimated population of 1.3 million, including 125,000 children under-5 years of age in around 100 sub-places^1^ ^1^Sub-places are referred to section of township, suburb, smallholding, village, sub-village, ward or informal settlement (StatSA 2001) [13,14]. At the inception of the SaT-HDSS, baseline data collection was implemented in eight mostly non-contiguous sub-places/clusters of low socio-economic status and high under-5 mortality rates in Soweto. Under-5 mortality rate of at least 50 deaths per 1000 live births was a major criterion for choosing the selected clusters to ensure that sufficient deaths are recorded to accurately quantify child mortality causes. The selected clusters may not be representative of the entire Soweto. Other sub-places not selected in Soweto had under-5 mortality rate lower than 50 deaths per 1000 livebirths. Based on 2011 census data (Table 2), under-5 mortality rates for the selected sub-places/clusters range from 51 deaths (per 1000 live births) in Braamfishervile Extension 12, Phiri/Senoane, and Thulani, to 84 in Mofolo North.

**Table 2.** Selected sub-places/clusters in Soweto with under-5 mortality estimates

S/N	Clusters	Total population	Under-five mortality rate†		
1	Braamfishervile Extension 12	4,750	15,426	1,521	51
2	Emdeni	8,527	35,753	3,582	53
3	Mapetla	7,533	23,146	2,031	53
4	Meadowlands Zone 4	3,292	11,739	1,209	58
5	Meadowlands Zone 5	3,162	13,970	1,473	56
6	Mofolo North	2,910	13,014	1,296	84
7	Phiri/Senaone	7,732	30,212	1,422	51
8	Thulani	11,139	40,376	40,376	51
	**Total**	**49,045**	****183,636****	**17,166**	

**Source**: Statistics SA (2011); †Under-five mortality rates are per 1000 live births based on South Africa’s 2011 census

**Figure 1.**
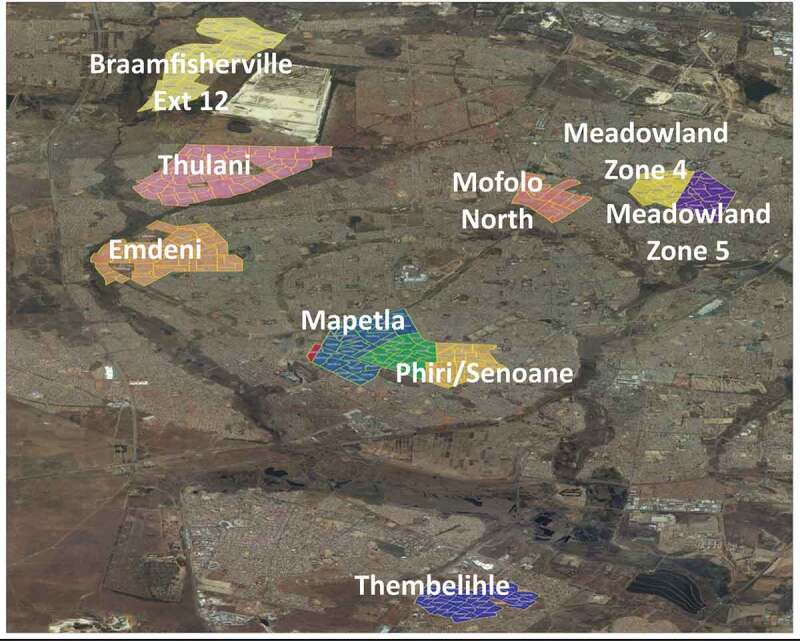
Map of Soweto/Thembelihle Demographic Surveillance Areas (DSA). The 8 clusters presented are sub-places in Soweto, with Thembelihle neighbouring Soweto

In mid 2018, the HDSS was extended to include Thembelihle – a contiguous informal settlement neighbouring Soweto (Figure 1). Population size and child mortality rates for these informal settlements were unavailable from South Africa’s statistical agency, because the settlements undergo continuous expansion; thus emphasising the need to routinely collect reliable data from this area. The informal settlements are mainly unplanned communities with limited access to basic services. Extension of the demographic surveillance area (DSA) was done in response to the high number of death notifications from Thembelihle and its surrounding areas.

The HDSS successfully completed baseline census in 2018. Subsequently it conducts 2 rounds of follow up household visits every year with 48 personnel, including fieldworkers and technical staff. Around 65% of the population in the selected sub-places has been successfully enrolled into the HDSS. The non-enrolled individuals represent approximately 35%, and this is due to unavailability of some participants and mainly because of boundary issues which are being resolved in some of the selected sub-places (Thulani, Braamfisherville and Emndeni informal settlements). A careful analysis and comparison of the enrolled individuals and the entire population of the study area show that the population currently under surveillance in the HDSS is representative of the selected sub-places in terms of population characteristics, composition and distribution.

The HDSS routinely collects information based on data elements as shown in Table 1. The HDSS is used for some on-going studies including mortality surveillance of under-5 children [11,12], diarrhoeal diseases among children, and household transmission of coronavirus disease 2019 (COVID-19).

### Data quality

SaT-HDSS has put data quality processes in place to ensure high quality and consistent longitudinal data. Our data collection system has in-built data consistency checks that identify inconsistent data. In addition, a team of quality control (QC) staff regularly conducts blinded repeat visits on 10% of total households randomly sampled across the DSA. Our analyses and comparisons of data from the regular interviews by fieldworkers and those from QC staff during our round 2 of follow up visit yielded a minimum of 98% consistencies across key demographic variables such as date of birth, date of death, pregnancy outcomes, survival status, and household size.

## Results

### Selected socio-economic and demographic information in SaT-HDSS

**Figure 2.**
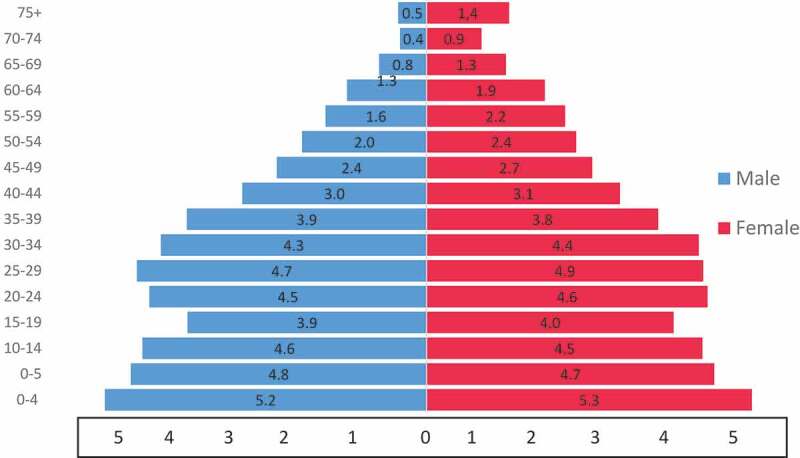
Pyramid of population under surveillance (SaT-HDSS 2019)

As presented in Table 3, the population currently under surveillance in the HDSS is 124,169 individuals in 31,779 households spanning 17.7 km^2^ in Soweto and 19.0 km^2^ in Thembelihle and surrounding informal settlements. The 8 sub-places (which are areas with high under-five mortality rates were selected based on the available and most recent census data – 2011 census). Although the plan was to enrol all individuals and households in the selected sub-places; however, not all individuals within the selected areas are easily accessible. As a result, there are individuals in the catchment areas that were not enrolled in the HDSS. Also, in-migration rate (defined as individuals having completed 4 calendar months after migrating into the DSA) stood at 2%, while out-migration rate in the DSA was 8% (Table 5). Our follow-up visits in rounds 1 and 2 yielded a 95% retention rate of the population enrolled at baseline. Figure 2 and Tables 2 and 3 further present the distribution of population currently under surveillance in SaT-HDSS.

**Table 3.** Population under surveillance in SaT-HDSS (December 2019)

S/N	Clusters	Number of households	Total population	Total under-5 population (N)
1	Braamfishervile Extension 12	2601	10,347	1226
2	Emdeni	4423	18,322	1806
3	Mapetla	2530	10,476	987
4	Meadowlands Zone 4	1562	7601	723
5	Meadowlands Zone 5	2290	9327	921
6	Mofolo North	2159	8347	816
7	Phiri/Senaone	4190	18,547	1817
8	Thulani	4313	17,939	1993
9	Thembelihle and surrounding informal settlements	7711	23,263	2659
**Total**		**31,779**	**124,169**	**12,948**

Table 4 presents the percentage distribution of selected socio-economic characteristics of the population under surveillance in SaT-HDSS. Majority of the population had secondary education and were unemployed. Further stratification of participants’ characteristics is as presented in Tables 4 and 5.

**Table 4.** Percentage distribution of the population under surveillance by selected socio- economic characteristics (SaT-HDSS 2019)

Variable	% of total population under surveillance (N = 124,169)
Male	48.0
Female	52.0
**Level of education (population aged 6+)**	
No formal education	11.4
Primary	22.5
Secondary (up to Grade 11)	34.9
Complete secondary (Matric)	24.0
Undergraduate (university)	2.5
Undergraduate (Tvet, Technical, etc.)	4.4
Postgraduate	0.3
**Employment (population aged 15+)**	
Not currently working	37.0
Full-time employed	24.7
Temporary/contract worker	7.6
Self-employed	6.1
Pensioner/retired	10.4
Student/Learnership	13.8
Home caregiver/Others	0.4
**Marital status (population aged 15+) above**	
Married, living with spouse	13.6
Married, not living with spouse	2.8
Living together with partner	15.7
Divorced	2.1
Widowed	5.5
Never married/never lived with partner	50.0
Currently single, but lived together with partner before	10.3
**Catchment area**	
Soweto	81.7
Thembelihle and surrounding informal settlements	18.3

**Table 5.** Some key demographic indicators in SaT-HDSS (2019)

Indicators	Soweto (N101,420)	Thembelihle (N = 22,749)	Total (N = 124,169)
Proportion of under-5 children to the total population (%)	8.4	10.9	8.7
Proportion of aged population (>65 years) to the total (%)	7.3	2.1	6.4
In-migration rate (% of individual population)	1.8	1.1	1.8
Out-migration rate (% of individual population)	7.8	4.5	7.9
Sex ratio at birth (%)	96.9	95.9	100.7
Average household size	3.7	4.0	3.0

## Discussion

The establishment of SaT-HDSS in a low-middle-income urban setting in Johannesburg, South Africa involved a number of challenges and lessons, which may inform future similar initiatives in other urban African settings.

### Challenges, mitigation approaches and lessons learned in setting up SaT-HDSS

#### 1. Community mobilisation and entry

Although our research unit has been working since 1998 (as an entity of the University of the Witwatersrand) in Soweto and its adjoining townships conducting biomedical studies, setting up the HDSS was faced with a lot of difficulties regarding community mobilisation and entry. This was partly due to the primary objective of CHAMPS programme which focuses on adverse pregnancy outcomes, including stillbirths and under-5 mortality. The problems relating to sensitivity of child death reporting and grieving about dead children among parents posed some challenges at the inception of the HDSS. Death is a sensitive issue to talk about in the African settings, it was therefore necessary to understand specific cultural, religious and socio-behavioural factors that may decrease or increase acceptability of CHAMPS surveillance procedures in the communities. Other challenges that we encountered in the process of community mobilisation included managing community dynamics; political prioritisation and conflicting agendas among political office holders that contradict the scientific aim of the HDSS; and managing community expectations such as monetary benefits for participating in the study.

Our findings from the focus group discussion (FGD) sessions which were conducted to understand community dynamics and priorities showed that some participants reported that they had suffered exploitation and abuse from researchers in the past. Furthermore, there were participants who expressed immediate personal needs that cannot be met, and such expectations made participating in the study irrelevant to them. We used evidence from our FGD sessions to undertake strategic planning and rigorous community engagement activities within the HDSS areas. A detailed report from the larger FGD sessions is available and is being developed as a separate publication.

### 2. Stakeholders’ engagement and participation

We encountered challenges regarding mistrust and misunderstanding about the study among many key stakeholders in the community. To address this problem, we conducted a number of stakeholder and community engagement activities, including intensive community awareness campaigns with various stakeholders and groups within the community. For instance, between August 2017 and July 2018, 28 workshops on Participatory Inquiry and Community Knowledge for CHAMPS (PICK CHAMPS) were conducted to provide a framework from which HDSS data collectors and other CHAMPS staff can learn about community priorities, values and responses to childhood illness or mortality (the report of PICK CHAMPS is available and is being developed for another publication). The CHAMPS programme has been able to establish good working relationships with many relevant stakeholders who now support HDSS to have access to households in the study catchment areas. These stakeholders include Ward Councillors, Ward Committee members, Street Committees, relevant government departments, private sector and civil societies, as well as relevant non-governmental organisations (NGOs) working on issues about children in Soweto and Thembelihle. Also, partnership was formed with organisations that work on issues that align with CHAMPS objectives.

### 3. Inaccessibility and concerns about safety of fieldworkers

During our first round of follow up visits, we had a substantial number of inaccessible households (>12,000, representing 48% of total number of households enrolled at baseline). To address this problem, we conducted FGD sessions to understand the reasons responsible for a high proportion of difficult-to-reach households. Some of the reasons adduced by FGD respondents included participants’ fear of strangers due to high crime rates in the community; fieldworkers’ fear about insecurity due to same reason of heightened criminality in the community; unavailability of employed participants during the normal working hours; communication gaps between our community engagement team and key community stakeholders about the timing of data collection; violence and substance abuse engendered by high rate of unemployment; concerns about safety of children (as children are often locked inside if parents are not around); fear of strangers among the elderly who live alone; fear of pets (such as security dogs) among fieldworkers; and difficult access to gated dwelling units with high walls. Another reason for inaccessible households cited by participants was the issue of participants who default in paying water and electricity bills. As a result, participants were unwilling to open their gates for strangers because our data collectors were mistakenly regarded as staff of municipal authorities that move around to enforce utility bill payment.

Our mitigation strategies against the problem of inaccessible households and safety concerns included partnering with key community stakeholders to provide safety supports for our field team; re-training of fieldworkers on how to ask sensitive or difficult questions such as child death with empathy and in a culturally acceptable manner; adjusting working hours for data collectors from 08:00am-04:00pm (Mondays-Fridays) to 10:00am-06:00pm during summer and 09:00am-05:00pm during winter (Tuesdays-Saturdays), including Sundays and Public Holidays on many occasions; organising regular community meetings; massive community outreach and campaigns through loud hailing across the length and breadth of the DSA; as well as strict compliance with the use of common uniforms and name tags by data collectors. Other implemented activities included clinic campaigns; cleaning awareness; fun walks, road shows, media campaigns on radio and newspapers, use of a Community Advisory Board (CAB), street committee members, ward councillors and community policing forum to reach the inaccessible households, and use of volunteers in the community to increase the visibility of the project.

The impacts of our strategic planning and stakeholders’ engagement were later felt as we had increased household access within the DSA. For instance, during our fieldwork and revisits to the hard-to-reach households which took place at the end of our first round of follow visit, the number of inaccessible households drastically reduced from over 12,000 to around 1300, thus leading to 95% retention of the total households enrolled at baseline.

### 4. Longitudinal nature of the HDSS and research fatigue

The longitudinal nature of the HDSS presented some challenges. Our data collection teams make repeated visits to a community that has several on-going research activities from many research units of the University of the Witwatersrand. This led to exacerbation of the already existing research fatigue in the community. Soweto is a highly researched area and community members tend to feel overwhelmed by all the research teams that continually come into their households for research-related activities. To address this challenge, our strategies were grounded on the principles of community engagement, as they demonstrate the importance of understanding communities as well as establishing trusting, respectful, equitable, and committed relationships. We organized a CAB with representatives from among the political, religious, traditional and other key community leaders within the study catchment areas. We extensively consulted with critical stakeholders such as Councillors who serve as gate-keepers in the community. We engaged with various stakeholders in order to understand community’s religious beliefs, cultural norms, political conditions, economic conditions, disease prevalence/incidence and environmental factors that may affect community buy-in and participation in the study.

### 5. Recruitment of technical and field staff

The recruitment of field staff was faced with less difficulty because there were many applicants with required qualifications; nevertheless, it was pretty difficult to get technical staff, such as demographers, computer scientists, data managers and programmers who have requisite experience. To address this problem, competent research and technical staff were recruited and were sent on a learning visit to some well-established HDSS sites, including the MRC/Wits––Agincourt HDSS, Mpumalanga, South Africa; African Health Research Institute HDSS, Kwazulu-Natal, South Africa; African Population and Health Research Centre HDSS, Nairobi, Kenya; Ouagadougou HDSS, Burkina Faso; and Kisumu HDSS, Kenya. Some of the important lessons that the technical staff received from the learning visits included (i) setting up an efficient structure for effective HDSS implementation; (ii) designing good data collection instruments; (iii) database set up and data management; (iv) effective field operation management; and (v) quality control to ensure good quality data. Of all the HDSS sites visited, only the Nairobi site is urban-based. Our experiences setting up an urban HDSS are quite different compared to the lessons from rural sites and some of these differences are presented in this paper.

### 6. Setting up HDSS database

With respect to setting up an appropriate HDSS database, an evaluation of different HDSS database systems and their potential use was done based on our organisation’s staffing level and cost of implementation. During the learning visits, it was observed that some HDSS sites were implementing in-house customised/developed database systems; however such customisation requires specialized skills. The decision to use the Open Health and Demographic System (OpenHDS) was then made because it was the only available system that could be deployed in a HDSS without any additional customisation. The INDEPTH Network– – which is the global network of health and demographic surveillance systems, also recommends the use of OpenHDS. Briefly, the OpenHDS is an open source application powered by Android. Its introduction was funded by Canadian International Development Agency, and was implemented by a number of partners––University of Calabar, Nigeria; the Swiss Tropical Institute, Switzerland; the Ifakara Health Institute, Tanzania; and the School of Public Health, Columbia University, USA. This application is useful for collecting individual level health and demographic data such as births, deaths, migrations, pregnancy observations and outcomes, immunisation histories and other important health and demographic information. OpenHDS application can be used for data collection offline; however, uploading the data to the OpenHDS server requires internet connectivity [15].

Despite the usefulness of OpenHDS, there is limited documentation and no technical support is available from its vendors for implementation of the system in a new site. This presented a great deal of challenge for a timely launching of SaT-HDSS. Each time we encountered technical problems during set up; there was no support from the provider. Attempts were made to contact them for assistance, but this was done to no avail. Due to a very limited documentation and lack of support from the developers, most of our implementations were done by trial and error until we found what worked. This was time-consuming and greatly affected the timely initiation of the HDSS. Moreover, because our implementations were largely through a process of experimentation, we implemented the system on incompatible data collection devices (tablets) that often crashed when fieldworkers were collecting data in the field.

Some of the technical glitches encountered were fixed through technical support from other HDSS sites. Moreover, as it is the case with longitudinal data collection, it is customary to prepopulate information collected at baseline for a follow up visit to avoid asking the same questions over and over again. Unfortunately, the core OpenHDS system is limited in this regard as it only processes and pushes very basic demographic information (such as age and sex) into the data collection devices for a follow up visit. To address this problem, we improvised a system of getting additional data directly into the tablet storage for the use of data collectors during the follow-up visits. It is an important lesson in setting up HDSS database that, although open source systems are freely available, they are often laced with technical glitches that require dedicated supports.

### Strengths and limitation of SaT-HDSS

As the first urban-based HDSS in the whole of Southern Africa, the site has a number of strengths. First, researchers and public health planners can triangulate the HDSS data with those from rural sites in South Africa and in the sub-region to generate evidence on mortality, burden of disease and other pertinent information that can guide efforts towards improving population and public health. Second, the SaT-HDSS can serve as a veritable platform for pilots of interventions and programmes aimed at improving health and well-being of the urban population such as was done in Soweto during the outbreak of COVID-19 (the platform was used for COVID-19 household transmission study, and screening and testing for COVID-19). In addition, data from the HDSS can be useful to support the research and career development of graduate students in relevant fields of population and public health. The limitation of SaT-HDSS concerns the selection of high under-5 mortality sub-places as catchment areas, hence the DSA may not be representative of the entire Soweto population. It covers low and middle income population of Soweto and Thembelihle where under-5 mortality is relatively higher compared to the rest of the township.

## Conclusion

Our experiences in setting up SaT-HDSS showed that the challenges of setting up an urban-based HDSS and rural sites differ significantly. The strategies we implemented led to a successful implementation. The challenges encountered in setting up the HDSS and the approaches and strategies we employed in addressing them may be found useful in setting up similar platforms in comparable settings. Some of the lessons learned while setting up the SaT-HDSS which could be applied in similar future endeavours include the need for a vigorous community mobilisation, continuous stakeholders’ engagement, as well as the need to ensure fieldworkers’ safety. Additionally, SaT-HDSS was initially set up as a component of CHAMPS programme, albeit it is being expanded to serve other studies. Thus, the criterion of selecting high under-5 mortality sub-places as catchment areas may be avoided in other future projects.

## Data Availability

The datasets generated in the HDSS can be made available on reasonable request and in line with the study protocol and funder’s terms and conditions.

## Paper context

SaT-HDSS has enrolled 124,169 individuals with 95% follow up rate of this population over a 2-year period. The HDSS has served as research platform for mortality surveillance, diarrhoeal diseases and COVID-19 household transmission study. Despite the challenges of setting up the HDSS, application of different strategies led to its successful takeoff. The HDSS offers important lens to providing evidence on broader population and public health issues in low-middle income settings.
